# Effects of Post-Annealing on the Properties of ZnO:Ga Films with High Transparency (94%) and Low Sheet Resistance (29 Ω/square)

**DOI:** 10.3390/ma16196463

**Published:** 2023-09-28

**Authors:** Li-Wen Wang, Sheng-Yuan Chu

**Affiliations:** Department of Electrical Engineering, National Cheng Kung University, Tainan 700, Taiwan; jk220052@gmail.com

**Keywords:** gallium zinc oxide, hydrogen, sheet resistance, transparency, annealing, XPS, SIMS

## Abstract

This study presents gallium-doped zinc oxide (ZnO:Ga, GZO) thin films. GZO thin films with both high transparency and low sheet resistance were prepared by RF sputtering and then post-annealed under nitrogen and hydrogen forming gas. With post-annealing at 450 °C, the proposed films with a film thickness of 100 nm showed high transparency (94%), while the sheet resistance of the films was reduced to 29 Ω/square, which was comparable with the performances of commercial indium tin oxide (ITO) samples. Post-annealing under nitrogen and hydrogen forming gas enhanced the films’ conductivity while altering the thin-film composition and crystallinity. Nitrogen gas played a role in improving the crystallinity while maintaining the oxygen vacancy of the proposed films, whereas hydrogen did not dope into the thin film, thus maintaining its transparency. Furthermore, hydrogen lowered the resistance of GZO thin films during the annealing process. Then, the detailed mechanisms were discussed. Hydrogen post-annealing helped in the removal of oxygen, therefore increasing the Ga^3+^ content, which provided extra electrons to lower the resistivity of the films. After the preferable nitrogen/hydrogen forming gas treatment, our proposed films maintained high transparency and low sheet resistance, thus being highly useful for further opto-electronic applications.

## 1. Introduction

Some monitors are composed of back luminous devices, such as OLEDs with transparent electrodes made from transparent conductive oxide (TCO) materials. The most common material in TCOs is indium tin oxide (ITO). However, efforts are being made to find alternative materials with low resistivity and high transmittance to replace ITO because of the high cost of indium and the diffusion problem [1,2]. As the device becomes smaller, the electrode thickness must be as thin as possible to maintain low resistance. Zinc oxide-based materials are good candidates because they are low-cost and non-toxic. Given that the conductivity of pure zinc oxide is not adequate for the desired purposes, group III elements, such as aluminum and gallium, are used as doping materials to provide extra electrons [3]. Compared with the covalent bond length of Zn–O, the variations in the covalent bond length of Al–O and Ga–O are 0.13 and 0.05 Å, respectively. Thus, gallium-doped zinc oxide (ZnO:Ga, GZO) has a smaller lattice deformation than aluminum-doped zinc oxide (ZnO:Al, AZO). Additionally, Ga is less reactive to oxidation than Al during the deposition. Therefore, GZO is more suitable than AZO to replace ITO. GZO thin films can be deposited using several methods, including the sol–gel method [4], atomic laser deposition [5], and sputtering [6]. This work prepared GZO thin films using RF sputtering due to their good thin-film adhesion and crystallinity. In general, zinc oxide-based TCOs have higher resistance than ITOs. Therefore, several methods to improve the conductivity of zinc oxide-based films have been suggested in previous studies, including the insertion of zinc oxide buffer layers [7], rapid thermal annealing (RTA) [8], and laser annealing [9]. TCO films post-annealed under hydrogen gas have been reported but with reduced transmittance [10]. Transmittance is an important point for transparent conductive materials. Researchers reported that the resistivity of zinc oxide films after argon and hydrogen forming gases post-annealing decreases but at the cost of transmittance [11].

Hydrogen gas is well known to reduce the resistivity of oxide film. However, how hydrogen affects thin films has not been discussed in detail. When pure zinc oxide undergoes pure hydrogen post-treatment, the conductivity changes due to interstitial hydrogen and hydrogen trapped in oxygen vacancies acting as two shallow donors [12]. Another study revealed that, following hydrogen post-treatment, hydrogen exists in the interstitial and substitutional sites of the Zn–O bonding matrix and can function as a donor [13]. Following the reaction between hydrogen and the zinc oxide thin film, the increment in oxygen vacancies caused by the hydrogen contributes to a lower resistivity than the pristine sample [11]. AZO is a well-known zinc oxide-based material for which hydrogen is also used to reduce the resistivity [14]. The reduction in conductivity can be attributed to the oxygen vacancies, Al interstitials, and Zn interstitials. Following hydrogen post-treatment, some zinc atoms are also replaced by aluminum [15], as indicated by X-ray diffraction (XRD), thus contributing to the changes in conductivity [10]. Hydrogen post-treatment has also been used to improve the conductivity of GZO thin films [16,17]. However, most of the reported studies focused on a discussion of the effects of crystal grain size and oxygen vacancies on improving conductivity at the cost of film transparency. In this study, GZO films were post-annealed under hydrogen and nitrogen forming gases to improve their conductivity and maintain their transmittance. The low resistivity and high transmittance of GZO thin films were simultaneously achieved. Material analysis revealed that hydrogen played a different role in the proposed thin films. The mechanism underlying the low resistivity of the thin films was also investigated.

## 2. Materials and Methods

GZO thin films were deposited using RF magnetron sputtering on a Corning Eagles (New York, NY, USA) glass substrate at room temperature. The ceramics target was 3 wt.% gallium oxide (purity 99.95%) and 97 wt.% zinc oxide (purity 99.95%) made by GFE (Nürnberg, Germany). The glass substrates were sequentially cleaned with deionized water (DI water), detergent, DI water, isopropanol, ethanol, and DI water in an ultrasonic tank. A detergent solution was used to remove dust and oil from the surface of the substrate, and an organic solvent was used to remove the organic particles from the substrate. In the final step, we used a hot plate to evaporate the water. The chamber vacuum had a pressure of 2 × 10^−5^ torr maintained by a maglev turbo pump (Osaka Vacuum, Osaka, Japan). The vacuum played a role in the thin film’s resistivity due to the mean free path. The deposition parameter was 60 W for 30 min at 2 mtorr with 3 sccm argon at room temperature. The film’s thickness was around 100 nm, as measured by ultrahigh-resolution transmission electron microscopy (TEM). In the post-treatment process, a furnace was employed for 1 h with (1) nitrogen (100%) and (2) hydrogen (15%)/nitrogen (85%) mixed forming gas at temperatures ranging from 400 °C to 550 °C. The 100% nitrogen sample served as the reference for comparison. The GZO thin films were characterized by Hall measurement (HMS-3000, Ecopia, Anyang, Korea), X-ray diffraction (D2, Bruker, Billerica, MA, USA), time-of-flight secondary ion mass spectrometry (SIMS) (TOF-SIMSV, ION-TOF, Münster, Germany), X-ray photoelectron spectroscopy (XPS) (PHI 5000 Versa Probe, ULVAC-PHI, Chigasaki, Japan), UV/Vis spectrophotometry ((JEM-2100F CS STEM, JEOL, Tokyo, Japan), and ultrahigh-resolution transmission electron microscopy (JEM-2100F CS STEM, JEOL, Tokyo, Japan) to determine the effect of post-treatment on lowering the film’s resistivity. The samples were labeled according to gas and temperature. For example, HN450 refers to a hydrogen and nitrogen mixture annealed at 450 °C.

## 3. Results and Discussion

**Figure 1:**
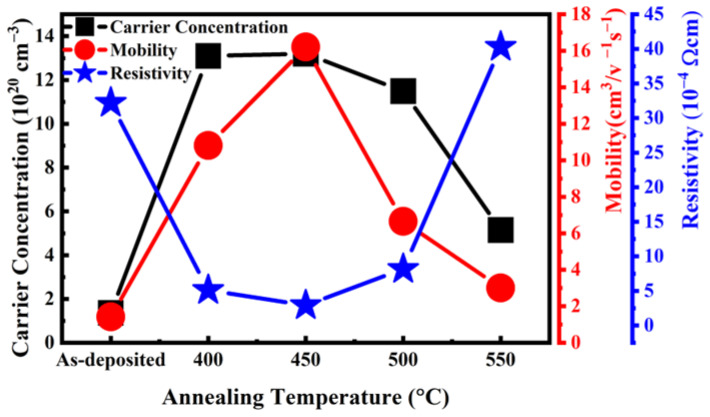
Carrier concentration, mobility, and resistivity of samples with and without forming gas post-treatment measured by Hall measurement.

Figure 1 shows the resistivity, carrier concentration, and Hall mobility for the samples with and without hydrogen forming gas post-annealing. The as-deposited samples had a resistivity of 3.22 × 10^−3^ Ω·cm, which is comparable with the performance shown in the reported paper [18]. The resistivity of the films decreased with the increase of the annealing temperature from 400 °C to 450 °C. The resistivity of the films reached the minimum of 2.9 × 10^−4^ Ω·cm at an annealing temperature of 450 °C and then increased again with a higher annealing temperature (>450 °C). The corresponding carrier concentration and mobility became the largest values that were compatible with or even better than the commercial ITO thin films. The resistivity of reference of commercial ITO was 1.97 × 10^−4^ Ω·cm with a thickness of 200 nm [19]. The best post-treatment sample had a resistivity of 2.9 × 10^−4^ Ω·cm with a thickness of 100 nm.

To determine the main reason the resistivity of the films decreased, pure nitrogen annealing at the same annealing condition was also conducted for comparison. Although an improvement in the crystallinity of the films under pure nitrogen annealing could be observed (as shown below), the carrier concentration and mobility of the films were worse in Hall measurements. Moreover, annealing in a pure nitrogen environment increased the resistivity of GZO thin films [20]. Therefore, hydrogen might play a major role during the annealing process to reduce resistivity in GZO.

Figure 1 shows that conductivity, σ = q (μ_n_N + μ_p_P), had a positive relationship with carrier concentration and mobility. In the equation, σ is the electrical conductivity, q is the electron charge, μ_n_ is the mobility of electrons, and μ_p_ is the mobility of holes. N is the electron carrier concentration, and P is the hole carrier concentration of the samples. As GZO is a zinc oxide doped material, the carrier concentration and mobility were both for the n type [21]. The conductivity is the inverse of resistivity. Given that σ is the conductivity, ρ is the resistivity. The equation is σ = 1/ρ [22]. The increase in mobility with increasing temperatures up to 450 °C was due to oxygen desorption at the grain boundary, which lowered the barrier potential [23]. The changes in carrier concentration and mobility were caused by the chemical reaction and the removal of oxygen [24] or the additional Ga^3+^ providing extra electrons [3].

**Figure 2:**
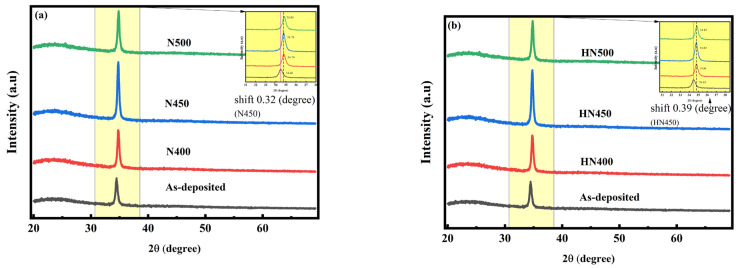
(**a**) XRD diagram of the hydrogen and nitrogen forming gas annealing thin GZO film (**b**) XRD diagram of GZO thin film annealed in pure nitrogen.

The annealing process may help to improve crystallization and change the electrical properties of thin films. Figure 2a,b shows the X-ray rocking curves for the as-deposited and annealed samples. In the XRD database, JCPDS card No. 361451, the polycrystal zinc oxide material has multipeaks in (002), (100), (101), and (103) [14,20]. Figure 2a,b also demonstrates that only the main peak (002) at 34° orientation (c-axis) was observed, and no other obvious peaks were observed probably due to the texturing effect [25]. No other secondary phases were related to Zn, Ga, or related compounds because the Ga ions might be incorporated into Zn positions in the hexagonal lattice as a dopant [25,26,27].

The other degrees of XRD in Figure 2a,b are flat, which means that the film was highly textured, and the crystals grew with the same preferred orientation. As the main peak (002) was amplified, the peaks were easy to observe after the annealing shifted to a higher degree for all annealed samples. The main peaks in Figure 2a slightly shift to a higher degree. In Figure 2b, the main peaks shift to a higher degree and are more evident. With the main peak XRD (full width at half maximum, FWHM) calculation result in Table 1, the Scherrer equation is D = K/cos θ, where D is the grain size, K is the shape factor, θ is the Bragg angle, and λ is the X-ray wavelength determined by the incident Cu (K = 0.154060 nm) source. Table 1 shows that the grain size increase due to annealing is the most popular way to increase crystallinity and reduce accumulated strain energy [3]. With the post-treatment, for either nitrogen or hydrogen forming gas, the grain size changed. It shows that the grain size of the samples after nitrogen and hydrogen annealing gas was the largest and the grain size of the pristine samples without any treatment was the smallest. Hence, post-treatment is the key to improving the grain size and FWHM of the samples. The XRD shifts in the forming gas-annealed sample might be deduced by atom replacement. Zn^2+^ and Ga ^3+^ atomic radii were 0.74 Å and 0.54 Å, respectively. This higher XRD shift indicates the lattice distortion caused by the strain. The strain might be induced by the substitution of Ga^3+^ for Zn^2+^. The smaller atomic radius of Ga^3+^ (0.54 Å) than Zn^2+^ (0.74 Å) led to a decreased lattice constant. The smaller radius of Ga^3+^, with a higher ion mobility than Zn^2+^, made Ga^3+^ more likely to combine with oxygen in an oxygen-deficient atmosphere [28]. The substitution of Ga^3+^ to Zn^2+^ caused the lattice constant to change, thus making the XRD shift higher in mixture gas annealing.

**Figure 3:**
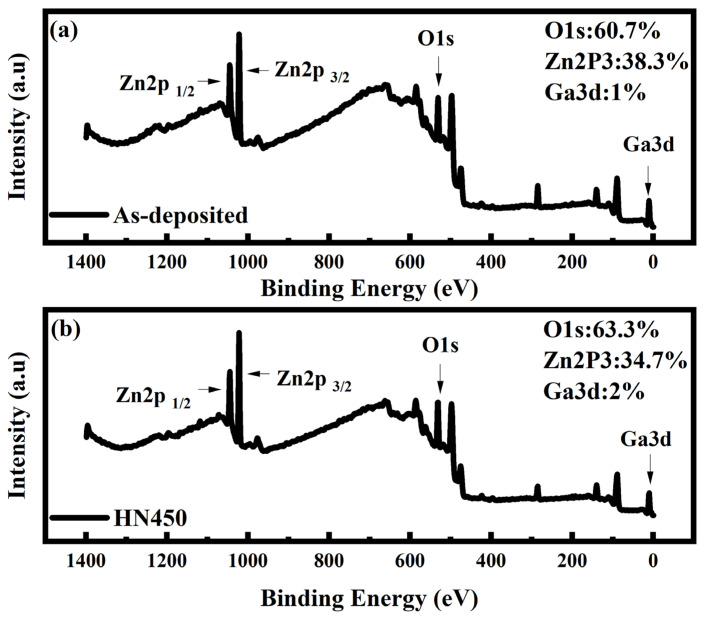
XPS spectrum (**a**) as-deposited sample; (**b**) mixture gas sample at 450 °C.

**Figure 4:**
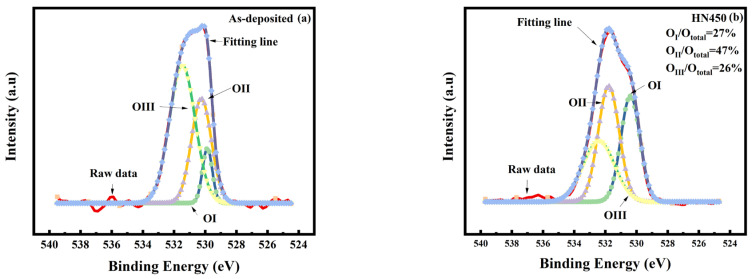
(**a**) XPS spectrum of OIs for the sample as it was deposited. (**b**) The HN450 sample’s XPS diagram is an OI configuration.

For the XRD analysis, all the samples annealed under forming gas (HN400–HN500) and pure nitrogen (N400–N500) exhibited better crystallization and a higher peak shift. However, the conductivity of these two samples differed from each other. Thus, crystallization might not be the major reason for conductivity improvement. X-ray photoelectron spectroscopy (XPS) was used to obtain information about thin-film elements and their chemical bonds. In the XPS wide scan energy spectrum shown in Figure 3a,b, the thin-film composition changed after forming gas annealing. From the XPS data in Figure 3, we found that the composition of Zn decreased from 38.3% to 34.7%, oxygen increased from 60.3% to 63.3%, and Ga increased from 1% to 2%. The total composition of the samples was 100%. Therefore, more gallium replaced the zinc inside the thin film after forming gas annealing. This outcome corresponds to previous XRD shifting results. As such, the resistivity should be reduced. As the focus on the XPS spectrum of O1s in Figure 4 shows, the O1s spectrum can be separated into three different peaks with fitting: OI, OII, and OIII. OI is the oxygen lattice, OII is the oxygen vacancy related to conductivity, and OIII is the surface oxygen, most of which is from the environment [29]. The hydrogen and nitrogen mixture gas provided anaerobic environments better than pure nitrogen annealing. The oxygen vacancy was one of the reasons for to reduced conductivity. In the previous Hall measurement in Figure 1, the conductivity was reduced the most at an annealing temperature of 450° C in mixture gas. Comparing the XPS O1s fitting diagram shown in Figure 4, the OII, which is related to oxygen vacancies, increased after forming gas annealing. The main reason oxygen vacancies contributed to the GZO thin film can be written as Vo↔Vo^2+^ + 2e^−^ [30]. An increase in oxygen vacancies provided additional electrons to increase the conductivity and carrier concentration. A comparison in Figure 4 shows that the oxygen interstitial also increased after annealing. The oxygen interstitial defects between the oxygen bonding with other atoms were another reason to improve the conductivity [31]. The annealing temperature provided the energy for the oxygen ions to detach from their normal space, which caused more defects to occur, thus improving conductivity. The energy provided by annealing might dissociate the bonding between atoms, which could cause atomic recombination.

**Figure 5:**
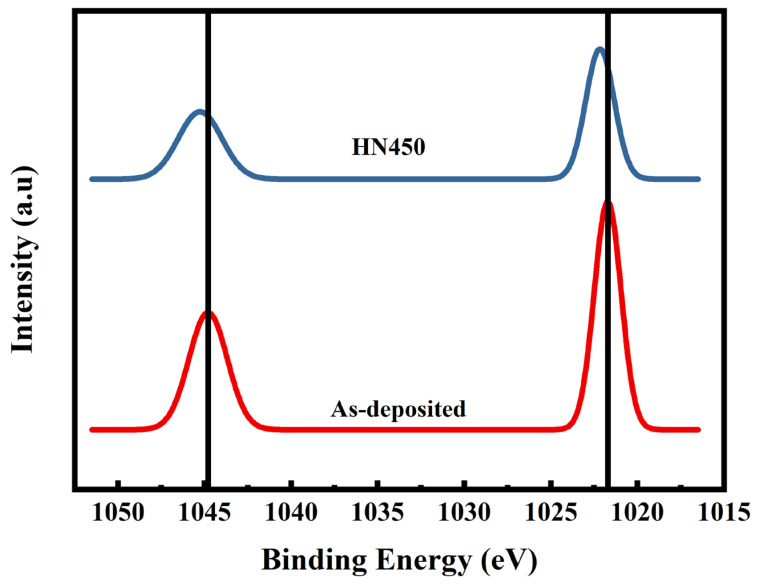
Spectrum of Zn 2p by XPS.

From the XRD results, the main peak (002) shifting to a higher degree indicates an increase in the shorter chemical bonds of the Ga^3+^ replacement of Zn^2+^. The XRD main peak shifts were caused by the doping concentration when the doping concentration was greater than ppm. The different doping concentrations with lattice expansion led to the XRD peak shifts [19]. OIII reduced drastically after gas annealing because most of the oxygen on the surface, combined with hydrogen, became water molecules and evaporated in the air. The desorption of oxygen on the surface caused the depletion region. Combining the Hall measurement results and the XRD analyses showed that the samples under HN450 post-annealing had the lowest resistance. In the Zn 2p XPS in the Gaussian fitting diagram shown in Figure 5, the peak with HN450 forming gas annealing shifted to the higher energy side, which means it became closer to the conductive band [32]. The Zn 2p XPS signal shifted, which would cause the binding between Zn and oxygen to become much weaker. In addition, more Zn was replaced by Ga, and more oxygen vacancy was found, which is in agreement with the SIMS results that will be shown later. The binding positions 1022 eV and 1044 eV attributed to Zn 2p1/2 and Zn 2p3/2, respectively, showed the zinc oxidation state (+2) [33]. The binding energy became lower, and the oxygen ions combined with the hydrogen became water molecules and evaporated during the process due to the high energy.

**Figure 6:**
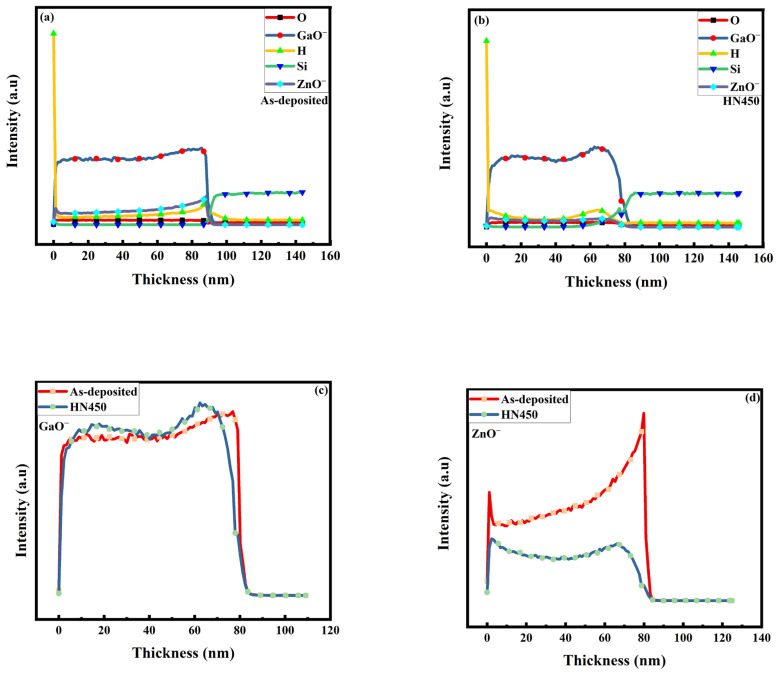
SIMS profiles for the sample (**a**) as-deposited; (**b**) for the 450 °C hydrogen and nitrogen forming annealing, (**c**) GaO^−^ and (**d**) ZnO^−^.

**Figure 7:**
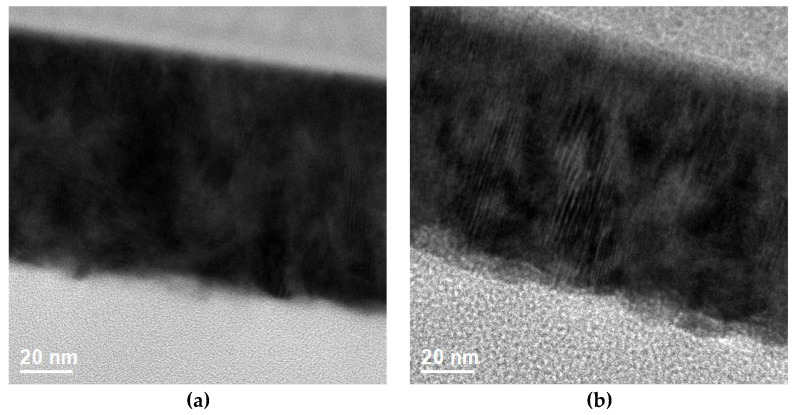
Cross-section of (**a**) as-deposited; (**b**) HN450 samples by TEM.

To determine how the hydrogen works after the annealing process, a secondary ion mass spectrometry (SIMS) depth profile can be used to detect the existence of hydrogen. This approach is more sensitive to element concentration (ppm) than XPS (%). The analysis diagram shows that the hydrogen intensity was similar for the pristine and annealed samples. Comparing Figure 6, the zinc intensity decreased after annealing, and the gallium intensity increased, thus being consistent with the XRD peak shift and the XPS spectrum results discussed previously. The intensity of the hydrogen inside the sample did not change substantially, which means that most of the hydrogen came from the sputtering deposition because hydrogen could only be vacuumed over an ultra-high vacuum. As a result, our proposed films retained good transparency (shown in Figure 7) after the post-annealing treatment, which is an unusual outcome compared with the findings of previous studies. Hydrogen from forming gas only affected the thin film’s surface, not as a doping agent inside the film. This result is consistent with the XPS data (OIII decreasing). Some of the zinc was replaced with gallium in the GZO thin film because gallium provides one more electron than zinc. Hence, the conductivity was reduced. With the SIMS diagram in Figure 6c,d, the intensity of the GaO^−^ ion increased. Meanwhile, the intensity of ZnO^−^ reduced after forming gas annealing in Figure 6d. This reduction meant that the trivalent gallium replaced zinc in the GZO host lattice [30]. After hydrogen forming in the post-treatment, the GaO^−^ increased, and additional Ga atoms were activated to generate carrier electrons from amorphous GZO grains, such as GaOx clusters [34].

**Figure 8:**
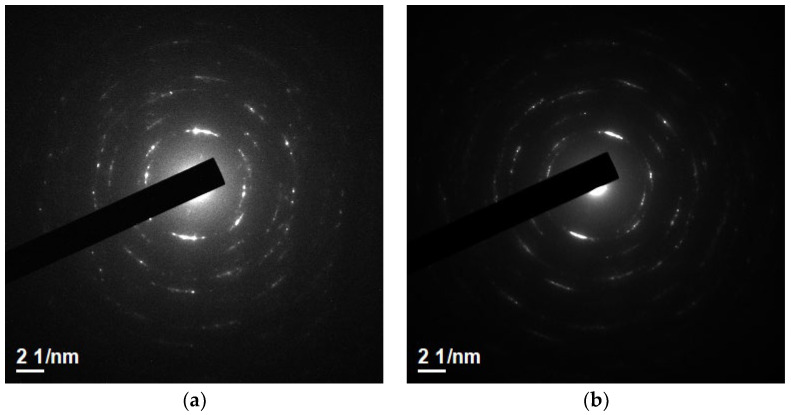
Diffraction pattern of (**a**) as-deposited; (**b**) HN450 samples by TEM.

Given that the thickness and crystallinity also affect the properties of thin film, the thickness was measured by TEM. The cross-section in Figure 7a,b shows that the thickness of the proposed films was around 100 nm. The thickness was not reduced after forming gas post-treatment. In the TEM diffraction pattern shown in Figure 8a,b, the crystallinity improved after post-treatment. Both diffraction patterns of the films were polycrystalline. Compared to previous XRD data, both films had only one peak because it was not grazing incident diffraction.

**Figure 9:**
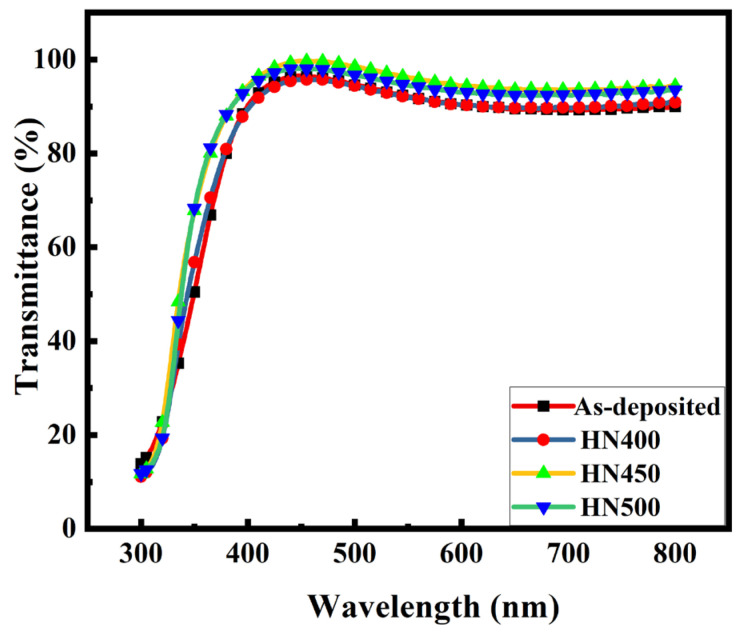
Transmittance with and without forming gas post-treatment samples.

**Figure 10:**
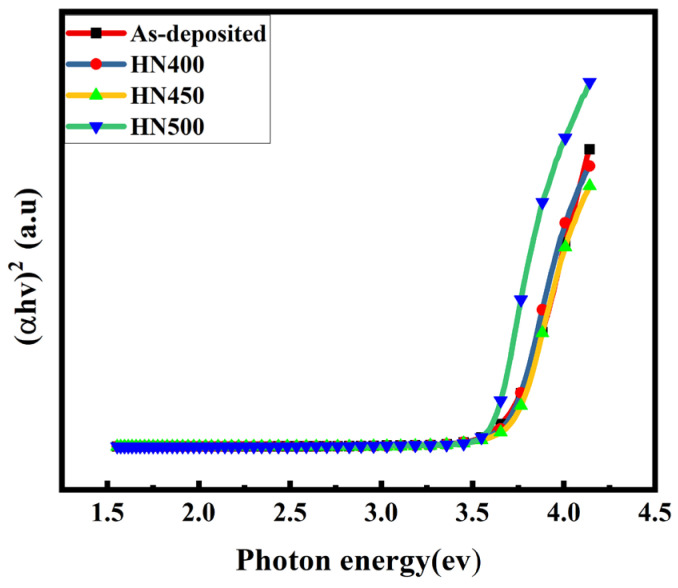
Optical band gap of the various gas-annealed GZO samples.

Figure 9 shows the transmittance of the as-deposited and hydrogen and nitrogen forming gas post-annealed samples. It shows that the transmittance improved even after annealing. The transmittance for all the samples was above 90% from 400 nm to 800 nm. The samples with 450 °C post-treatment (HN450) achieved the best transmittance (over 95%) in the visible spectrum, which is good for TCO. These results are much better than the ITO thin film with the same deposition method by PVD on a glass substrate with a thickness of 200 nm, a sheet resistance of 32 Ω/square, and a transmittance of over 84% [35]. These improvements in transmittance have several causes. One is the carrier concentration, and another is the crystallinity. When the carrier concentration increases, the interstitial defects in the thin film decrease [36]. In this case, the thickness reduction shown in the SIMS result is the other reason for better transmittance. The XRD analyses present better crystallinity with the narrower FWHM after annealing post-treatment, where the transmittance increased due to the improved crystallinity [37]. Another reason is that the GZO thin films with the mixture annealing treatment did not have extra doping inside the films that would have affected the transmittance [8]. Zinc oxide-based materials have favorable optical properties, where the band gap can be calculated using optical absorption, as shown in Figure 10. The bandgap was deduced using Tauc’s equation = (αhν)1/n = A (ην − Eg), where α, h, and ν refer to the absorption coefficient, the Planck constant, and the radiation frequency, respectively. A and η are constants. Eg is the energy gap shown in Table 2. The HN450 sample had the largest energy gap value of 3.7 eV. The band gap can change for several reasons. An increase in the carrier concentration is due to the Burstein-Moss effect [38]. The other reason is that when the Ga concentration increases, the band gap increases as well [39], which is in agreement with experiments. According to the basic idea, a smaller bandgap means more conductivity. However, most references show zinc-oxide-based materials with hydrogen annealing after the optical band gap increases [11,40,41]. The band gap may not be the main reason for reducing conductivity.

**Figure 11:**
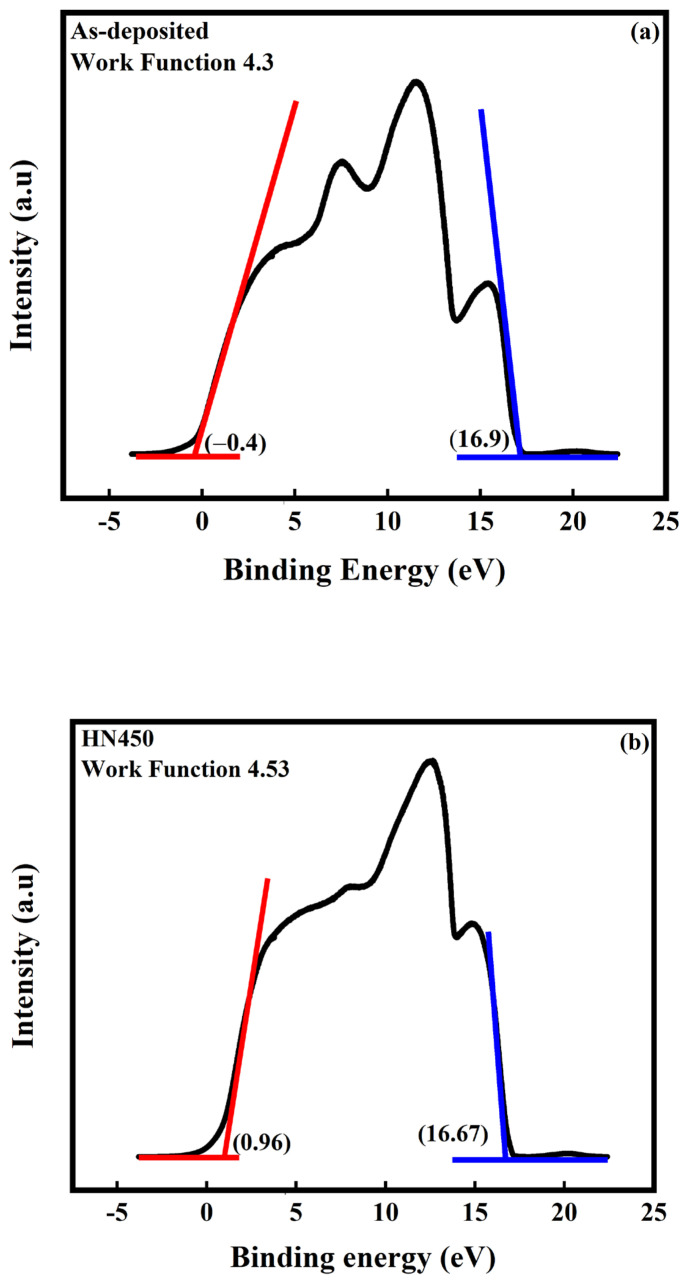
UPS spectrum of (**a**) as-deposited; (**b**) 450 °C mixture gas annealing sample. The blue line in the figure is used to obtain the cut-off of the high binding energy value for the calculation of the work function. The red line is used to obtain the low binding energy to obtain the value for the calculation of the valence band minimum.

**Figure 12:**
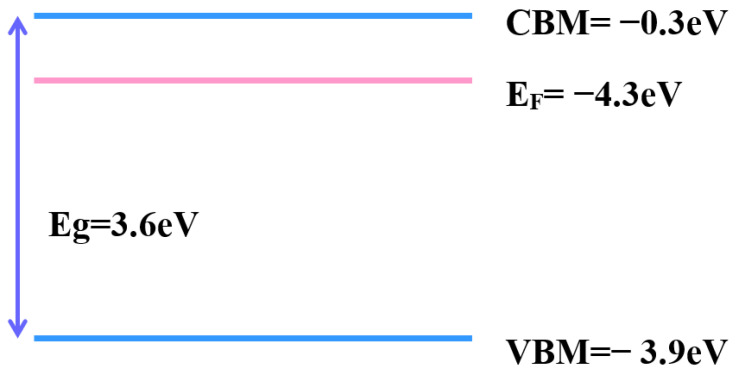
Band diagram of the as-deposited sample.

**Figure 13:**
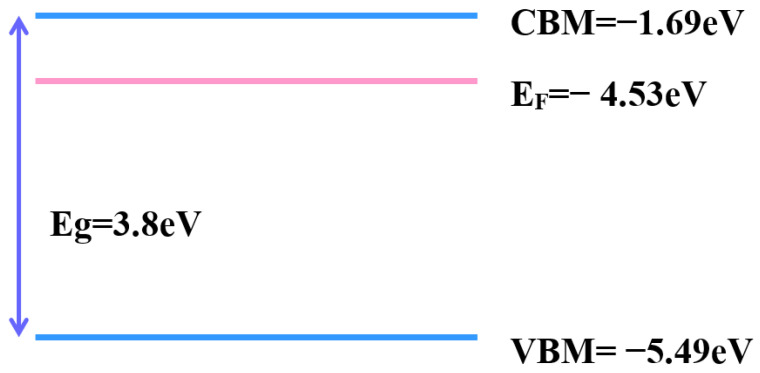
Band diagram of 450 °C annealing samples.

Tuning the work function provides a more effective application for transparent conductive oxide. The work function provides band alignment in electronic and optical devices, which may be one reason to improve device performance. Ultraviolet photoelectron spectroscopy (UPS) is a popular way to measure work function (Figure 11a,b). The work function of as-deposited is 4.3 eV, while that of mixture annealing is 4.53 eV. A previous report [42] revealed that the work function of zinc oxide-based TCO decreases when oxygen vacancies are filled. By contrast, the increment in oxygen vacancies makes the work function larger. The other reason for the change in work function is the doping concentration [43]. The trivalent element increased, and the work function value had a positive correlation. With the UPS spectrum, the conduction band minimum (CBM), valence band maximum (VBM), and position of Fermi level (E_F_) are determined, as shown in Figure 12 and Figure 13. With this parameter, the whole band gap diagram can be achieved. The band gap of zinc oxide was 3.2 eV to 3.57 eV [44], and the band gap of gallium oxide was 4.58 eV [45]. The band gap between 3.2 eV and 4.58 eV was reasonable.

## 4. Conclusions

This study achieved the low sheet resistivity and high transparency of GZO thin films prepared by RF sputtering. This study found that hydrogen and nitrogen forming gas annealing post-treatment is a useful way to improve the conductivity of GZO thin films. The material analyses indicated that the changes in thin-film composition and oxygen vacancies are the main reasons for the improvement in conductivity. The annealed temperature can help to improve crystallization and accelerate the chemisorption of hydrogen combined with surface oxygen, which increases oxygen vacancies. According to the SIMS and XPS analyses, gallium composition increased after the post-treatment. By contrast, the zinc composition decreased. Some of the zinc was replaced by gallium, which provided additional electrons, thus reducing the resistance. After annealing, the hydrogen did not dope inside the thin film to maintain the same transmittance. After the annealing treatment, the GZO thin film became competitive or even better than the most commonly used ITO of transparent conducting thin films. Therefore, the proposed GZO samples have the potential for transparency in conducting thin-film applications.

## Figures and Tables

**Table 1:** Grain diameters of samples annealed with pure nitrogen, hydrogen, and nitrogen.

Sample Name	FWHM (Degree)	Grain Size (nm)	Sample Name	FWHM (Degree)	Grain Size (nm)
As-deposited	0.54	14.02	As-deposited	0.54	14.02
HN400	0.46	16.20	N400	0.53	15.30
HN450	0.45	16.85	N450	0.52	15.80
HN500	0.47	16.12	N500	0.54	15.01

**Table 2:** Photon energy of the GZO samples.

	As-Deposited	HN400	HN450	HN500
Photon energy (eV)	3.6	3.6	3.7	3.4

## Data Availability

Not applicable.
